# Potentially compromised systemic and local lactate metabolic balance in glaucoma, which could increase retinal glucose and glutamate concentrations

**DOI:** 10.1038/s41598-024-54383-4

**Published:** 2024-02-14

**Authors:** Mina Arai-Okuda, Yusuke Murai, Hidetaka Maeda, Akiyasu Kanamori, Takako Miki, Tomoko Naito, Kazunobu Sugihara, Michihiro Kono, Masaki Tanito, Hiromitsu Onoe, Kazuyuki Hirooka, Yoshiaki Kiuchi, Masakazu Shinohara, Sentaro Kusuhara, Sotaro Mori, Kaori Ueda, Mari Sakamoto, Yuko Yamada-Nakanishi, Makoto Nakamura

**Affiliations:** 1 Division of Ophthalmology, Department of Surgery, Kobe University Graduate School of Medicine, 7-5-2 Kusunoki-cho, Chuo-ku, Kobe, 650-0017 Japan; 2 Maeda Eye Clinic, Osaka, Japan; 3 Kanamori Eye Clinic, Akashi, Japan; 4 Grace Eye Clinic, Okayama, Japan; 5 Department of Ophthalmology, Shimane University Faculty of Medicine, Izumo, Japan; 6 Department of Ophthalmology and Visual Science, Hiroshima University Graduate School of Biomedical Sciences, Hiroshima, Japan; 7 Division of Molecular Epidemiology, Department of Future Medical Sciences, Kobe University Graduate School of Medicine, Kobe, Japan; 8 The Integrated Center for Mass Spectrometry, Kobe University Graduate School of Medicine, Kobe, Japan

**Keywords:** Glaucoma, Molecular neuroscience, Neurodegeneration

## Abstract

To investigate the association between lactate metabolism and glaucoma, we conducted a multi-institutional cross-sectional clinical study and a retinal metabolomic analysis of mice with elevated intraocular pressure (IOP) induced by intracameral microbead injection. We compared lactate concentrations in serum and aqueous humor in age-matched 64 patients each with primary open-angle glaucoma (POAG) and cataract. Neither serum nor aqueous humor lactate concentrations differed between the two groups. Multiple regression analysis revealed that only body mass index showed a significant positive correlation with serum and aqueous humor lactate concentration in POAG patients (r_s_ = 0.376, P = 0.002, and r_s_ = 0.333, P = 0.007, respectively), but not in cataract patients. l-Lactic acid was one of the most abundantly detected metabolites in mouse retinas with gas chromatography and mass spectrometry, but there were no significant differences among control, 2-week, and 4-week IOP elevation groups. After 4 weeks of elevated IOP, d-glucose and l-glutamic acid ranked as the top two for a change in raised concentration, roughly sevenfold and threefold, respectively (ANOVA, P = 0.004; Tukey–Kramer, P < 0.05). Glaucoma may disrupt the systemic and intraocular lactate metabolic homeostasis, with a compensatory rise in glucose and glutamate in the retina.

## Introduction

Glaucomatous optic neuropathy (GON) is a chronic, progressive, degenerative disease of retinal ganglion cells (RGCs) and their axons within the optic nerve. While intraocular pressure (IOP) and aging play significant roles, genetic and environmental factors also contribute to the development and progression of GON. However, the specific mechanisms underlying these factors remain unclear^1^.

Lactate, once considered a byproduct of anaerobic metabolism, is now recognized to be produced even in aerobic conditions. It is transported from lactate-producing tissues to consuming tissues, serving as (1) a significant energy source replacing glucose, (2) a major precursor of gluconeogenesis, and (3) a signal transducer molecule^2,3^. In the central nervous system, blood glucose is taken up by astrocytes through the glucose transporter GLUT1, metabolized into lactate through the glycolytic system, and extracellularly released via monocarboxylate transporters (MCTs) 1 and 4. Neurons are believed to take up this released lactate through MCT2, convert it to pyruvate, and utilize it for ATP production through oxidative phosphorylation in the TCA cycle^4–7^. Both our research and others’ findings have indicated that optic nerve crush and high IOP in animal models may disrupt lactate transport by reducing MCT expression, potentially leading to RGC death^7–9^. These observations suggest that GON may involve disturbances in systemic or local ocular lactate transport and metabolism. In line with this concept, several previous studies have compared blood and aqueous humor lactate concentrations in human glaucoma patients to those in cataract patients. Vohra et al. reported that serum lactate levels in patients with normal tension glaucoma (NTG) were significantly lower than in controls^10^. However, Bouchemi et al. reported significantly higher plasma lactate levels in patients with primary open-angle glaucoma (POAG) compared to cataract patients^11^. Bouchemi et al.^11^ and Jovonavic et al.^12^ also found significantly higher lactate concentrations in the aqueous humor in patients with POAG than in those with cataracts. Thus, there is currently no consensus on whether there are differences in serum and aqueous humor lactate in glaucoma and cataract patients. Additionally, little is known about how lactate and related metabolites in the retina are altered in animal models of glaucoma.

This study aimed to (1) examine the association between serum and aqueous humor lactate concentrations and POAG by comparing those concentrations in POAG and cataract patients and to (2) perform a metabolomic analysis of the retina of a mouse model with elevated IOP to identify changes in the profile of retinal l-lactic acid, ionized form of lactate, and related metabolites in response to elevated IOP stress.

## Results

### Serum and aqueous humor lactate concentrations and their associated factors in patients with cataract or POAG

The background of involved patients is shown in Table 1. Age, height, weight, body mass index (BMI), systolic, diastolic, and mean blood pressure, casual blood glucose, ocular perfusion pressure, and exercise frequency per week did not differ between the cataract and POAG groups. Oxygen saturation was significantly lower in the POAG group. Intervals between the last meal and blood sampling were also slightly shorter in the POAG group. Corrected visual acuity was significantly worse in the cataract group. Spherical equivalent and central corneal thickness values were significantly lower, but IOP was significantly higher in the POAG group. In the cataract and POAG groups, 28 (43.8%) and 34 (53.1%) were male, 38 (59.4%) and 29 (45.3%) involved the right eye, and 30 (46.9%) and 32 (50.0%) were systemic drug users, respectively, with no significant differences between the two groups (chi-square test, P = 0.29, 0.11, and 0.73, respectively).

**Table 1:** Patient information.

	Control (n = 64)				Glaucoma (n = 64)				MWT
	Min	Max	Med	IQR	Min	Max	Med	IQR	P value
Age, years	52	84	72.5	66.8–77.0	50	86	70.0	64.0–74.0	0.15
Height, m	1.46	1.78	1.60	1.53–1.69	1.40	1.80	1.63	1.57–1.68	0.25
Weight, kg	34	90	55.0	49.8–65.0	32.5	96.4	58.6	48.8–66.1	0.73
BMI	15.5	33.1	22.2	20.4–23.6	12.7	32.8	22.0	19.9–23.8	0.66
SBP, mmHg	101	174	134.0	118.0–148.5	89	168	134.5	120.0–147.0	0.95
DBP, mmHg	46	103	77.5	66.0–86.3	56	114	78.5	71.8–86.5	0.18
MBP, mmHg	70	127	95.3	85.4–105.8	68	132	96.2	89.6–104.8	0.42
OPP, mmHg	29.3	78.9	50.3	42.6–56.5	32	68	47.8	43.3–52.9	0.18
SpO_2_, %	96	100	99.0	98.0–100	95	100	98.0	97.8–99.0	0.001
CBG, mg/dL	70	153	94.5	88.8–106.0	74	160	100	94–110	0.07
logMAR	0.00	1.00	0.22	0.15–0.30	− 0.18	0.82	0.10	0.00–0.22	< 0.001
SE, diopter	− 16.88	3.38	− 1.88	− 4.66–0.66	− 19.88	2.75	− 4.13	− 7.56– − 1.41	0.007
CCT, μm	452	645	550.0	521.5–580.0	448	638	525.0	496.0–550.3	< 0.001
IOP, mmHg	10	20	14.0	13.0–15.0	10	35	16.0	14.0–20.0	< 0.001
Exercise/week	0	7	1.5	0–2.5	0	7	1.0	0.0–4.1	0.65
Sampling Interval, min	120	855	270	220–312	60	937	225	174–280	0.01

*MWT* Mann–Whitney U test, *Min*. minimum, *Max*. maximum, *Med*. median, *IQR* interquartile range, *BMI* body mass index, *SBP* systolic blood pressure, *DBP* diastolic blood pressure, *MBP* mean blood pressure, *OPP* ocular perfusion pressure, *SpO*_*2*_ saturation of percutaneous oxygen, *CBG* casual blood glucose, *LogMAR* logarithm of minimal angle resolution, *SE* spherical equivalent, *CCT* central corneal thickness, *Exercise* number of 30-min or longer workouts per week that result in perspiration, *Sampling Interval* interval between the last meal to blood sampling.

Table 2 shows the serum and aqueous humor lactate and pyruvate levels for the cataract and POAG groups. No significant differences were found. Lactate and pyruvate were approximately 7.3 and 3.7 times higher in aqueous humor than in serum, respectively.

**Table 2:** Lactate and pyruvate concentrations in serum and aqueous humor in patients with cataract or POAG.

	Control (n = 64)				Glaucoma (n = 64)				MWT
	Min	Max	Med	IQR	Min	Max	Med	IQR	P value
Serum Lac, mg/dL	3.2	15.8	7.3	5.4–9.5	3.6	21	7.2	5.4–9.6	0.81
Serum Pyr, mg/dL	0.2	1.2	0.6	0.4–0.8	0.2	1.8	0.6	0.4–0.8	0.29
AH Lac, mg/dL	37.2	79.6	53.0	47.8–56.7	32.4	92.4	52.4	46.2–60.3	0.88
AH Pyr, mg/dL	0.4	4.8	2.2	1.8–2.6	0.2	4.2	2.2	1.8–2.8	0.69

*MWT* Mann–Whitney U test, *Min*. minimum, *Max*. maximum, *Med*. median, *IQR* interquartile range, *Lac* lactate, *Pyr* pyruvate, *AH* aqueous humor.

Table 3 presents the results of univariate and multivariate regression analyses of factors related to serum lactate concentration. Given that serum lactate is a glucose metabolite, blood glucose was excluded from the explanatory variables. Both in the univariate and multivariate regression analyses, only BMI showed a significant correlation. Table 4 displays the results of univariate and multivariate regression analyses of factors associated with aqueous humor lactate concentration. Blood glucose was included and intervals between the last meal and aqueous humor collection was excluded as an explanatory variable due to the different sampling dates for blood and aqueous humor. The univariate regression analysis revealed significant correlations with age, BMI, and spherical equivalent, while the multivariate regression analysis showed a significant correlation only with BMI.

**Table 3:** Univariate and multivariate regression analyses of factors possibly associated with serum humor lactate concentrations.

	Univariate			Multivariate		
	ß	95% CI	P value	β	95% CI	P value
Age	− 0.046	− 0.077, 0.045	0.607	− 0.034	− 0.083, 0.060	0.744
BMI	0.238	0.059, 0.363	**0.007**	0.256	0.061, 0.392	**0.008**
SpO_2_	0.043	− 0.309, 0.512	0.626	0.081	− 0.249, 0.627	0.394
Exercise	0.024	− 0.187, 0.245	0.791	0.035	− 0.176, 0.262	0.696
MBP	0.058	− 0.027, 0.055	0.512	0.026	− 0.036, 0.048	0.771
SE	0.084	− 0.055, 0.157	0.346	0.099	− 0.067, 0.187	0.354
Sampling interval	− 0.104	− 0.205, 0.053	0.244	− 0.145	− 10.324, 1.051	0.109
Disease	0.003	− 1.032, 1.070	0.972	0.037	− 0.920, 1.364	0.384

Significant values are in [bold].

*CI* confidence interval, *BMI* body mass index, *SpO*_*2*_ saturation of percutaneous oxygen, *Exercise* number of 30-min or longer workouts per week that result in perspiration, *MBP* mean blood pressure, *SE* spherical equivalent, *Sampling interval* duration between the last meal to blood sampling, *Disease* cataract or primary open-angle glaucoma.

**Table 4:** Univariate and multivariate regression analyses of factors possibly associated with aqueous humor lactate concentrations.

	Univariate analysis			Multivariate analysis		
	ß	95% CI	P value	β	95% CI	P value
Age	0.214	0.049, 0.448	**0.015**	0.177	− 0.026, 0.437	0.081
BMI	0.204	0.095, 1.123	**0.021**	0.241	0.178, 1.259	**0.010**
BS	0.172	− 0.001, 0.202	0.052	0.067	− 0.065, 0.143	0.458
SpO2	− 0.128	− 2.367, 0.368	0.151	− 0.024	− 1.630, 1.248	0.793
Exercise	0.05	− 0.520, 0.932	0.575	0.051	− 0.489, 0.913	0.551
OPP	− 0.110	− 0.302, 0.069	0.216	− 0.089	− 0.279, 0.090	0.312
SE	0.289	0.244, 0.928	** < 0.001**	0.169	− 0.075, 0.758	0.107
Disease	0.003	− 3.461, 3.592	0.971	0.036	− 2.992, 4.420	0.704

Significant values are in [bold].

*CI* confidence interval, *BMI* body mass index, *SpO*_*2*_ saturation of percutaneous oxygen, *Exercise* number of 30-min or longer workouts per week that result in perspiration, *OPP* ocular perfusion pressure, *SE* spherical equivalent, *Disease* cataract or primary open-angle glaucoma.

**Figure 1:**
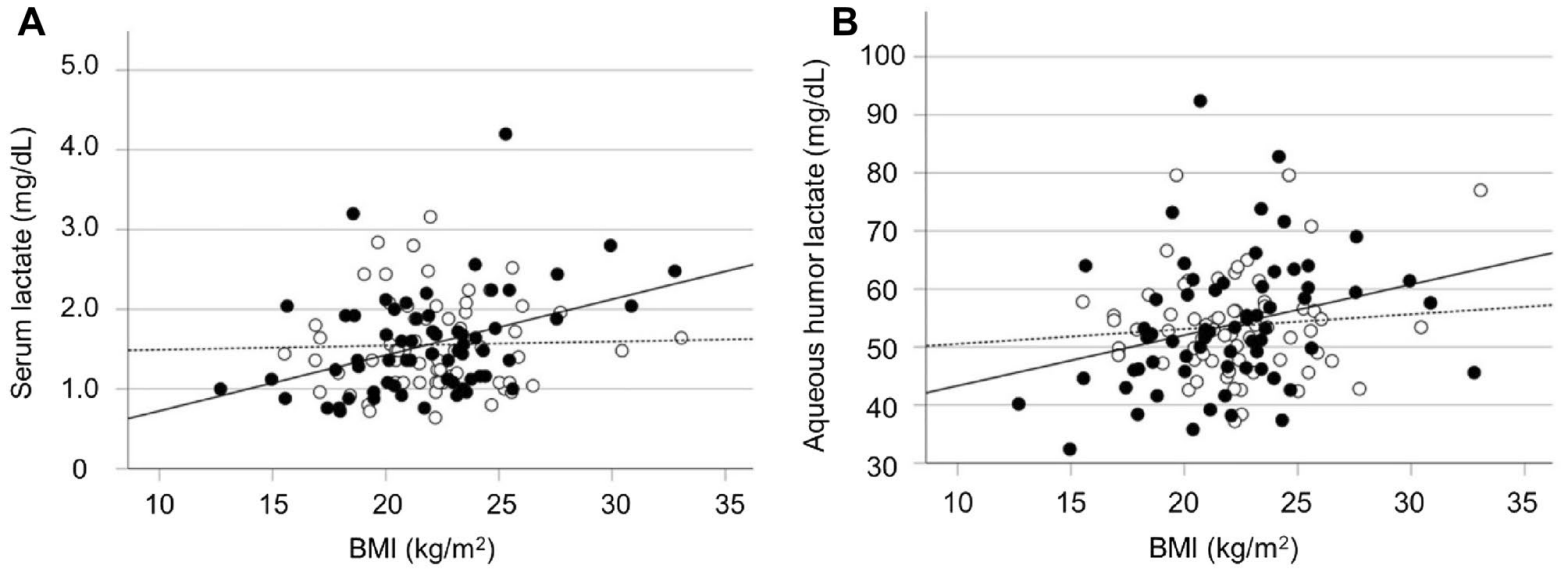
Association between body mass index (BMI) and lactate levels in serum and aqueous humor. Open circles and dotted lines represent patients with cataract, whereas closed circles and solid lines represent patients with primary open-angle glaucoma (POAG). (**A**) Relationship between BMI and serum lactate concentration. Cataract group, r_s_ = 0.028, P = 0.827. POAG group, r_s_ = 0.376, P = 0.002. (**B**) Relationship between BMI and aqueous humor lactate concentration. Cataract group, r_s_ = 0.042, P = 0.745. POAG group, r_s_ = 0.333, P = 0.007.

Figure 1 illustrates the relationship between BMI and serum and aqueous humor lactate concentration for the cataract and POAG groups. In the POAG group, a significant positive correlation was observed between lactate and BMI for both serum and aqueous humor (r_s_ = 0.376, P = 0.002 and r_s_ = 0.333, P = 0.007, respectively). However, in the cataract group, there was no significant correlation between the two in either sample (r_s_ = 0.028, P = 0.827 and r_s_ =  − 0.042, P = 0.745, respectively).

### IOP changes in mice with intracameral microbeads injection

**Figure 2:**
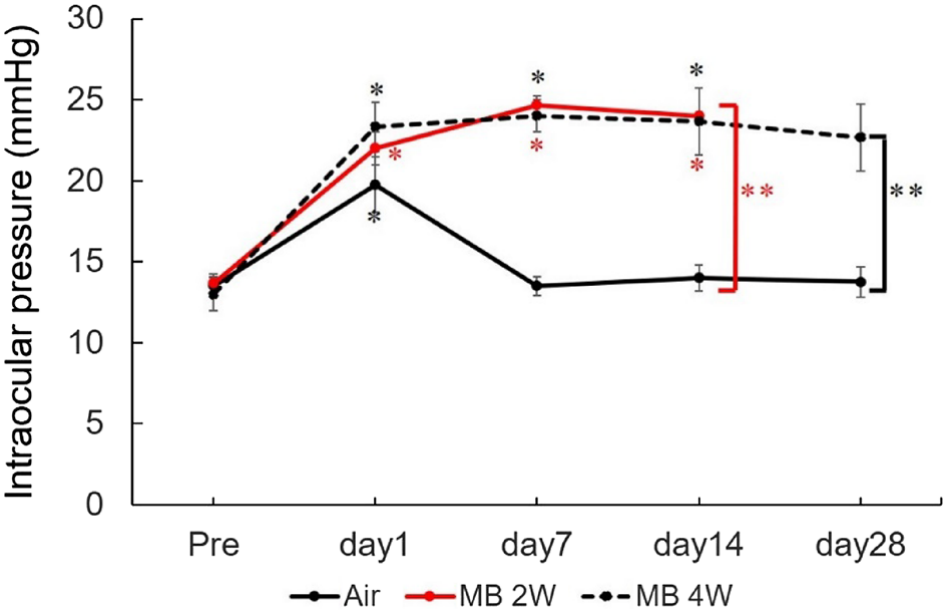
Changes in intraocular pressure (IOP) over time in mice with elevated IOP models following intracameral microbead injection. Air, air bubble-injected control group (n = 4); MB 2W, 2-week microbead-injected group (n = 3); MB 4W, 4-week microbead-injected group (n = 3). Pre, before injection. Error bar denotes standard deviation. *Significant rise compared to pre-treatment (Friedman test; P values are shown in the text). **Significant rise compared to air bubble-injected control group at respective time points (unpaired t-test; P values are shown in the text).

Figure 2 shows the variations in IOP in mice over a 4-week period of air injection and 2–4 weeks of microbead injection. The initial IOP levels before treatment were 13.5 ± 0.6 mmHg in the air injection group, 13.7 ± 0.6 mmHg in the 2-week microbead injection group, and 13.0 ± 1.0 mmHg in the 4-week microbead injection group. There was no significant difference among the groups (ANOVA, F = 0.7, P = 0.53).

In the air injection group, IOP was significantly higher at 1 day post-treatment compared to pre-treatment (Friedman, F = 5.15, P = 0.012), but afterward, it did not significantly differ from pre-treatment levels. In the 2-week microbead injection group, IOP at all time points after treatment was significantly higher than pre-treatment (F = 82, P = 0.00003). In the 4-week microbeads group, IOP was significantly higher at 1, 7, and 14 days post-treatment than pre-treatment (F = 4.33, P = 0.037). The trend was also elevated at 28 days of treatment, but the difference was not statistically significant. The IOP in the 2-week and 4-week microbead injection groups was significantly higher than that in the air injection group at the same time (t = 14.64, P < 0.0001 and t = 7.73, P = 0.0006, respectively). This IOP trend was consistent with our previous study^9^.

### Retinal metabolite profile changes induced by elevated IOP

**Figure 3:**
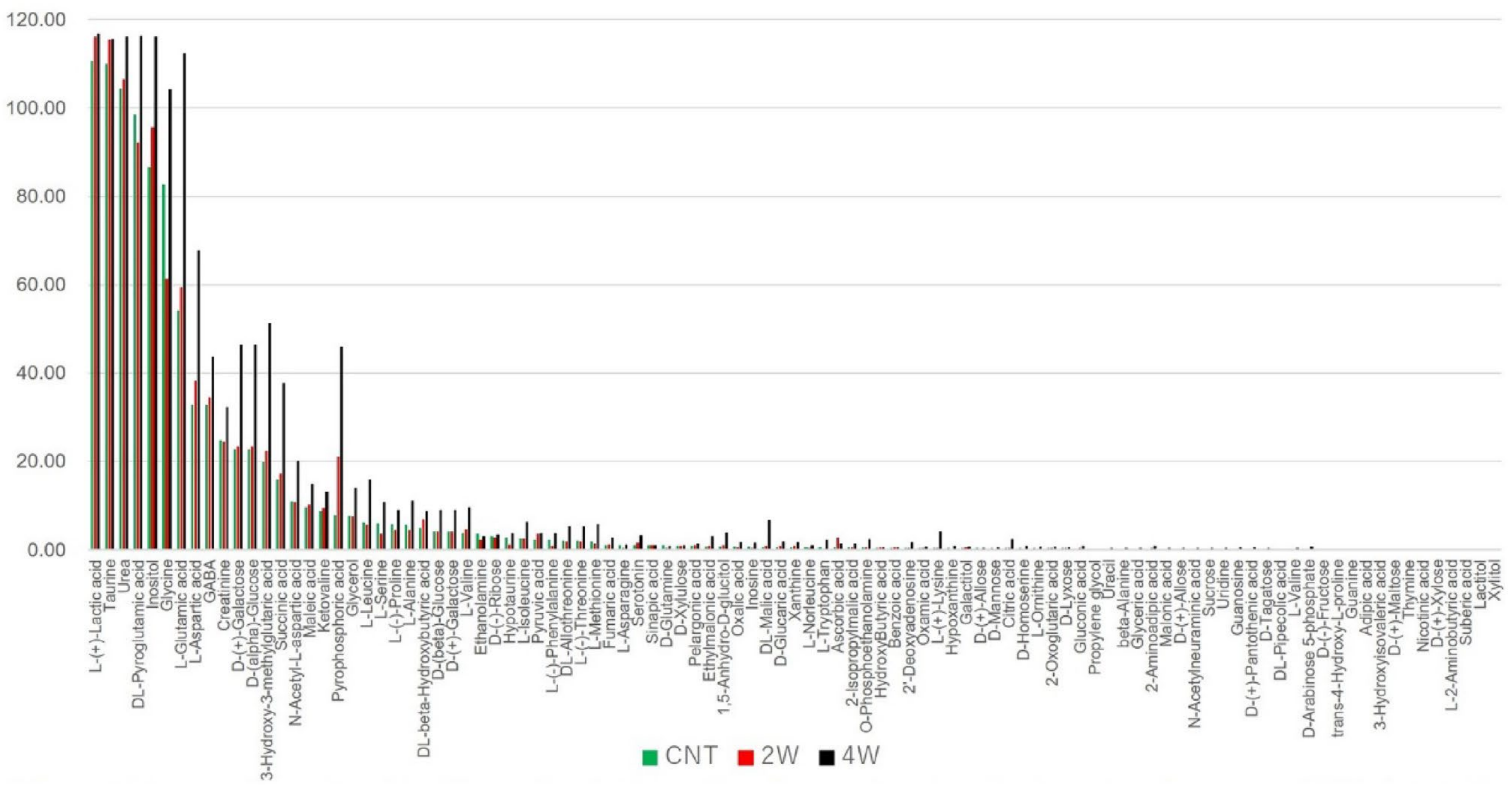
Comparison of metabolite profiles in the retina of control and ocular hypertensive mice. CNT, air bubble-injected control eyes (n = 4). *2W* 2-week microbead-injected eyes (n = 3). *4W* 4-week microbead-injected eyes (n = 3). Metabolites with a relative expression ratio greater than 2.0 compared to sinapinic acid are shown in median values. They are arranged in descending order based on their expression levels in the control group.

Figure 3 shows the average retinal metabolites for the 4-week air-injected (control), 2-week microbead-injected, and 4-week microbead-injected groups. The metabolites are arranged in order of increasing expression, with a relative ratio to sinapinic acid greater than 2.0 in the control group. l-Lactic acid, the ionized form of lactate, was one of the most abundantly detected metabolites, along with taurine and urea. There was no significant difference in the l-lactic acid concentrations among the groups (ANOVA, F = 0.05, P = 0.95; F = 0.045, P = 0.96; F = 0.13, P = 0.877).

**Figure 4:**
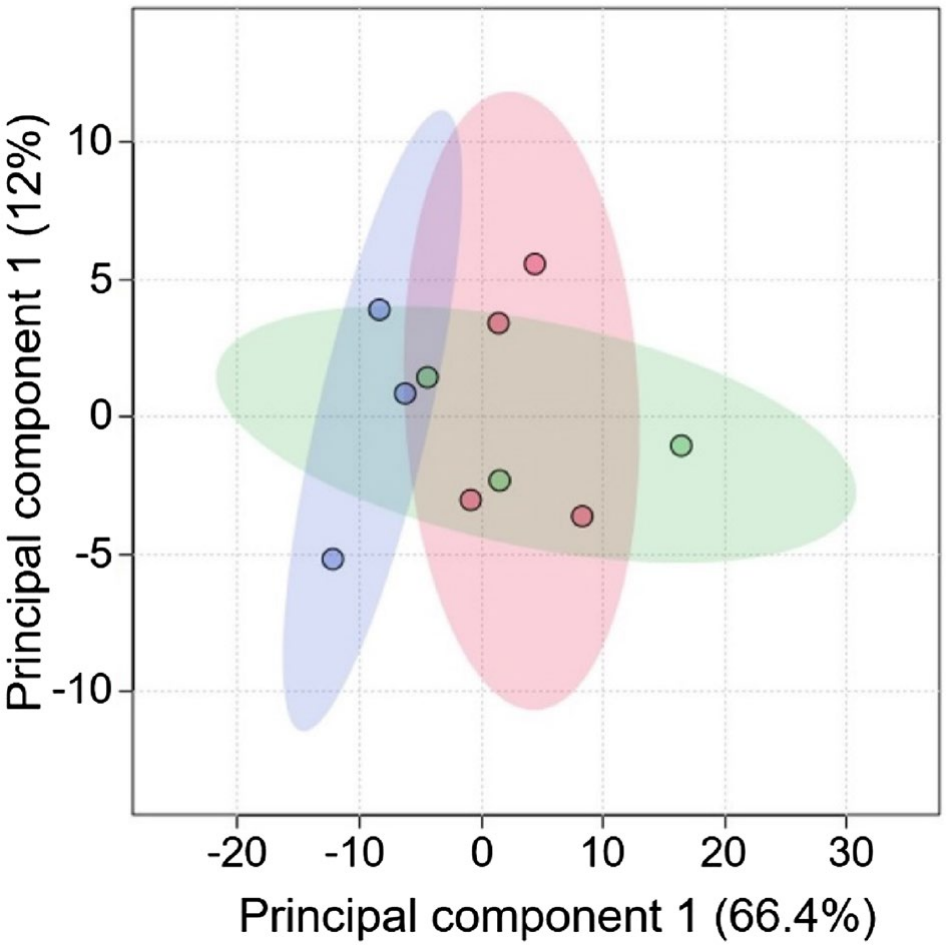
Two-dimensional plots derived from metabolomic principal component analysis. Red circles and ellipse, air bubble-injected control. Green circles and ellipse, 2-week microbead-injected group. Blue circles and ellipse, 4-week microbead injection group. Whereas ellipses show a 95% confidence interval of the identical treatment groups, circles show each sample.

**Figure 5:**
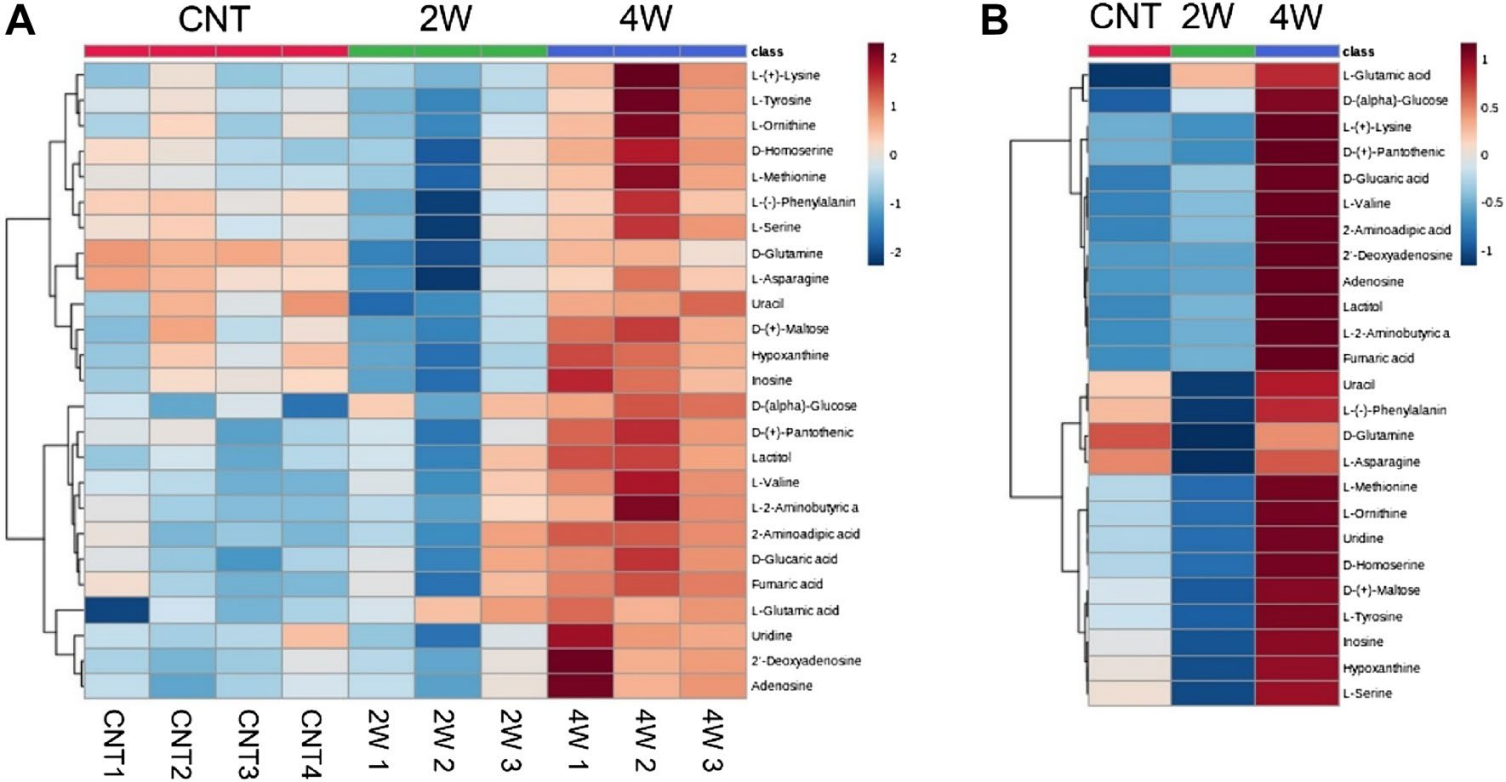
Top 25 mouse retinal metabolites that differ in expression across groups, as shown by hierarchical cluster analysis. CNT, air bubble-injected control eyes (n = 4). *2W* 2-week microbead-injected eyes (n = 3). *4W* 4-week microbead-injected eyes (n = 3). (**A**) Individual-based analysis results. (**B**) Results of analysis based on group means.

**Figure 6:**
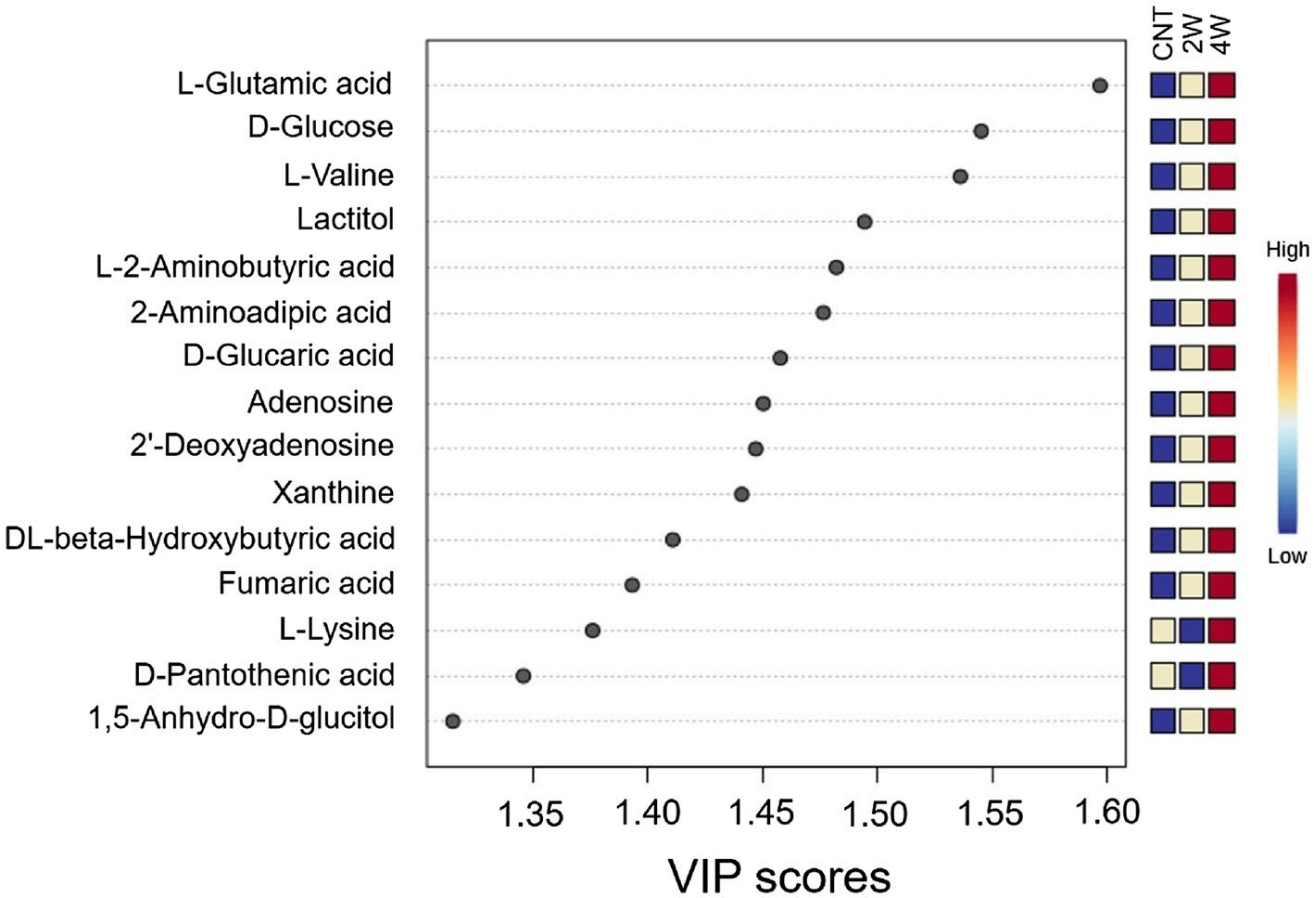
Variable importance in the projection (VIP) scores of mouse retinal metabolites analyzed by partial least squares-discriminant analysis. Metabolites with VIP scores greater than 1 are shown. CNT, air bubble-injected control eyes (n = 4). *2W* 2-week microbead-injected eyes (n = 3), *4W* 4-week microbead-injected eyes (n = 3).

**Figure 7:**
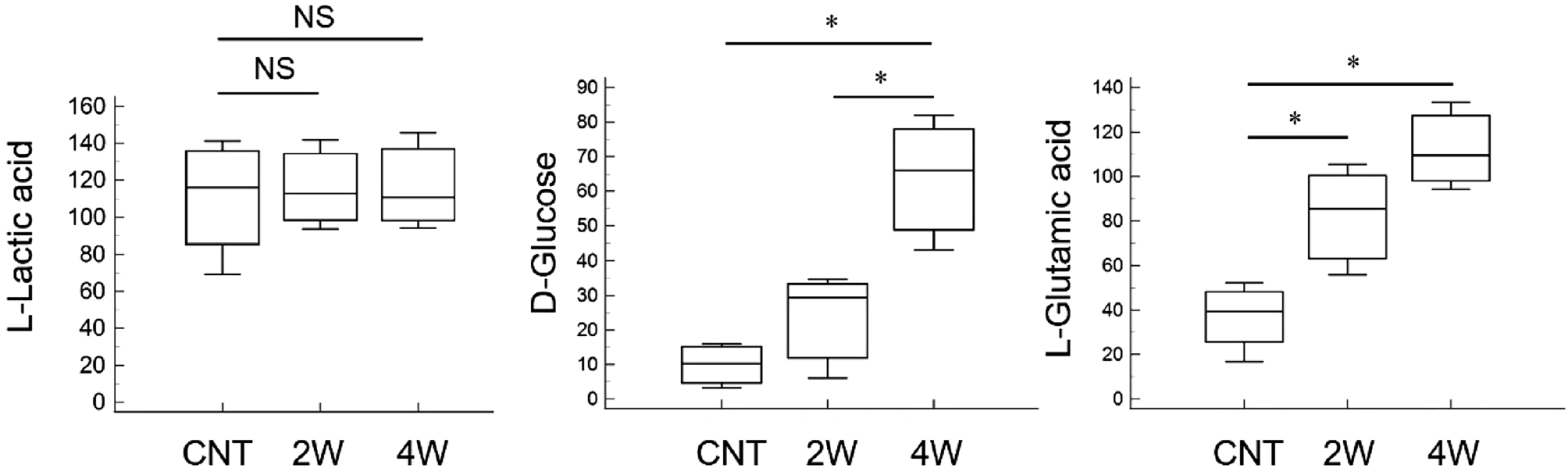
Box-and-whisker plots of the relative ratios of l-lactic acid, d-glucose, and l-glutamic acid in retinas in three different conditions. CNT, air bubble-injected control eyes (n = 4). *2W* 2-week microbead-injected eyes (n = 3). *4W* 4-week microbead-injected eyes (n = 3). P < 0.05 (Tukey–Kramer test).

Figure 4 shows the superimposed two-dimensional score plots of the principal component analysis for the three groups. No outliers were observed, and all groups displayed distinct distribution patterns. Hierarchical cluster analysis showed the top 25 metabolites with the most significant intergroup variation, both for each individual and the average of each group (Fig. 5). The analyses revealed distinct profiles of retinal metabolites among the three groups. Figure 6 shows the metabolites ranked in the top 15 variable importance in the projection (VIP) scores in partial least squares discriminant analysis (PLSDA), with l-glutamic acid and d-glucose obtaining the highest and second highest VIP scores, respectively. Figure 7 shows a box-and-whisker plot with relative ratios of the two metabolites, along with l-lactic acid, across the three groups. l-Lactic acid was present at high concentrations in all groups and did not exhibit significant differences (ANOVA, F = 0.05, P = 0.95). However, d-glucose was significantly higher in the 4-week microbead injection group compared to the 4-week air and 2-week microbead injection groups (ANOVA, F = 13.45, P = 0.004; Tukey–Kramer P < 0.05). l-Glutamic acid was also significantly higher after 2 and 4 weeks of microbead injection compared to the air-injected group (ANOVA, F = 12.98, P = 0.004; Tukey–Kramer P < 0.05).

## Discussion

No significant differences in serum and aqueous humor lactate and pyruvate concentrations were found between the two groups, each consisting of 64 patients with cataract or POAG with no history of metabolic syndrome-related diseases (i.e., diabetes or hyperlipidemia) other than hypertension. Resting serum lactate concentrations were reported to range from 0.33 to 1.67 mM^19,20^, similar to the present results (median for cataract patients = 0.81 mM, median for POAG patients = 0.80 mM). In both groups, aqueous humor lactate was more than seven times higher than serum lactate, similar to previous reports^21,22^.

In the metabolomic analysis of the mouse retina, l-lactic acid was one of the most abundantly detected metabolites in both the control group and 2- and 4-week IOP elevation groups. There were no significant differences among the three groups. Total retinal lactate levels in mammals were reported to fluctuate between 5 and 50 mM^23^, making the retinal lactate concentration considerably higher than that of serum and closer to that of aqueous humor. This finding aligns with the results of the current clinical study, where the median aqueous humor lactate concentration was 5.88 mM in cataract patients and 5.82 mM in POAG patients. The high lactate concentration in aqueous humor has been traditionally interpreted as an accumulation of lactate as a waste product due to glucose consumption in the cornea and lens^21,22^. However, considering that lactate is a cellular energy substrate that is sometimes preferred over glucose, and that tissues are equipped with MCTs responsible for transporting lactate along concentration gradients, the substantial differences in lactate concentrations between aqueous humor or retina and serum suggest that lactate might not solely serve as a waste product^24^. Instead, in the aqueous humor and retina, lactate appears to be intentionally metabolized and derived from glucose, implying a more functional role for lactate.

However, the results of the current clinical study, which indicated no difference in serum and aqueous humor lactate concentrations between the cataract and POAG groups, did not align with previous reports^10–12^. Vohra et al.^10^ reported that resting serum lactate in 11 healthy subjects and 12 patients with NTG, excluding those with hypertension, hyperlipidemia, diabetes, autoimmune disease, or a history of stroke, was 1.1 mM and 0.7 mM, respectively, with the latter being significantly lower. It is worth noting that our study did not exclude individuals with systemic hypertension and included not only NTG patients but also POAG patients with high IOP. The small sample size of the Vohra et al. study may also in part account for the discrepancy between their and our results. Bouchemi et al.^11^ also compared the plasma and aqueous humor concentrations of 114 cataract patients with those of 100 POAG patients. Based on their findings, plasma lactate concentrations were 1.74 mM in cataract patients and 2.55 mM in POAG patients, and aqueous humor lactate concentrations were 4.71 mM and 5.81 mM in the respective groups, with higher concentrations in the POAG patient group for both samples. Their study^11^ did not specify clear exclusion criteria, and the plasma lactate concentrations in both groups significantly exceeded the serum lactate levels of normal subjects at rest, as reported in other studies^19,20^. This suggests that there might be potential confounding factors contributing to fluctuations in lactate concentrations. Jovonavic et al.^12^ reported that the aqueous humor lactate concentration in 40 eyes of 30 cataract patients was 0.022 ± 0.03 U/L and that in 42 eyes of 30 POAG patients was 0.042 ± 0.04 U/L, which was significantly higher in POAG patients. However, due to their different measurement methods and units, direct comparison with other reports is not feasible. They also included data from both eyes of the same patient, potentially introducing bias. The sample size setting in our study was designed to detect differences in serum or aqueous humor lactate between cataract and POAG patients, and therefore, the results indicating no difference between the two groups should be considered exploratory data. To establish a non-inferiority design and ensure validation, more data need to be collected and analyzed.

In the present study, lactate concentrations in serum and aqueous humor were significantly positively correlated with BMI in both univariate and multivariate regression analyses, but only in POAG patients. These results suggest that cataract patients have a healthy homeostasis mechanism that keeps resting serum lactate concentrations within a certain range regardless of BMI, while POAG patients may lack this mechanism in a healthy state. This situation, in which stability is maintained through robust, energy-demanding adaptive mechanisms in response to various physical, mental, and environmental stresses, is referred to as allostasis^25^. The presence of serum and aqueous humor lactate concentration gradients correlated with BMI only in POAG patients may indicate that POAG patients are in an unhealthy adaptive response state of energy substrate metabolism in response to systemic and ocular local stresses. A high BMI, or obesity, may lead to attenuated lactate consumption by oxidative phosphorylation or an excessive glycolytic condition, while a low BMI, or being underweight, may be constantly exposed to the opposite condition. There is no consensus regarding the association between obesity, metabolic syndrome, and POAG. Both overweight and underweight have been reported to increase the risk of POAG^26,27^. For example, in the Tajimi study, no association was found between BMI and Japanese POAG patients and controls^28,29^. However, the majority of patients in the Tajimi study had NTG, whereas the present study included a significant number of POAG patients with high IOP, which may explain the disparity between the two results^30^. Even among individuals with similar degrees of obesity, often measured by BMI, clinical prognosis can vary depending on their overall metabolic status^31–33^. Obese but metabolically healthy individuals tend to have lower levels of insulin resistance, visceral fat, and cardiovascular event risk. In contrast, non-obese but metabolically unhealthy individuals are more likely to experience higher levels of insulin resistance, visceral fat, blood pressure, oxidative stress, and cardiovascular complications^32–36^. The study population consisted of POAG patients with at least one indication for cataract or glaucoma surgery, suggesting that they may have more advanced glaucoma and potential metabolic abnormalities, if not outright metabolic syndrome.

Mouse retinal metabolome analysis revealed that elevated IOP stress led to increased glucose and glutamate concentrations in the retina. In normal physiology, glucose concentrations in brain parenchyma are tightly regulated to remain at less than half of blood glucose levels^37^, similar to the aqueous humor. When blood glucose levels rise, the brain’s glucose uptake is significantly reduced to maintain normal metabolic levels^38^. Elevated brain glucose levels can also lead to increased glutamate concentrations^38^. Previous metabolomic analyses have shown that glucose levels in the retina increased approximately threefold 2 weeks after optic nerve crush^15^ and by over 50-fold in spontaneously developed glaucoma mouse retinas^16^. The possibility that excitotoxicity, induced by excess intracellular and extracellular glutamate, plays a role in optic neuropathy in glaucoma has long been suggested^39^. In various animal studies, including those conducted by our research group, optic nerve crush, elevated IOP, and spontaneously developed glaucoma models have been shown to reduce the expression of MCTs^7–9^. In response, compensatory increases in glucose transporter expression occur^7^. Therefore, at least relatively acute elevated IOP stress may reduce the efficiency of lactate transport, the preferred energy substrate of RGCs, and increase their dependence on glucose. Since glucose metabolism and the glutamine/glutamate cycle are interconnected^24^, an abnormal increase in glucose uptake may simultaneously lead to elevated glutamate production and excitotoxicity.

This study has several limitations. First, the inclusion of patients taking various medications raises the possibility that these medications may have influenced serum and aqueous humor lactate concentrations. However, there were no significant differences in the type and frequency of oral medications used in the cataract and POAG patient groups (data not shown). Second, nearly all glaucoma patients were using glaucoma eye drops. While it is possible that these eye drops could affect the metabolite profiles in the aqueous humor, it is likely that their impact on lactate metabolism in the aqueous humor is not significant, as there was no significant difference in median aqueous lactate concentrations between the cataract and POAG patient groups. In fact, a previous report showed that timolol eye drops did not affect oxygen consumption, glucose metabolism, or lactate production in the ciliary body root of rabbits^40^. Although oral acetazolamide medication could increase the aqueous humor lactate concentration^21^, only two glaucoma patients were taking oral acetazolamide in the current study, both of whom showed serum and aqueous humor lactate concentration within the IQR of the group values. Out of all the research we could find, there was no additional study that looked at the association between specific glaucoma medications and aqueous humor lactate metabolism. The slightly lower oxygen saturation in the POAG group suggests the potential impairment of oxidative phosphorylation in these patients. However, further investigation is needed to confirm whether oxygen saturation is indeed lower in POAG patients. Third, the degree of lens opacity may have affected the lactate concentration in the aqueous humor. Unfortunately, the detail is not known, because we did not record the grading of lens opacity in this study. Finally, the number of samples for metabolomic analysis of the mouse retina is also relatively small, and additional studies, including assessments of reproducibility, are needed.

In conclusions, there was a positive correlation between serum and aqueous humor lactate concentrations with BMI, but only in POAG patients. However, the median values were not different from those in cataract patients. Retinal l-lactic acid, one of the most abundantly detected metabolites, remained unchanged in the control and experimental models, while d-glucose and l-glutamic acid concentrations in the 4-week elevated IOP mice were significantly increased compared to the control group. Glaucomatous insults may systemically and locally impair lactate metabolism homeostasis, which may lead to glucose and subsequent glutamate overload in the retina.

## Materials and methods

### Subjects for clinical study

The clinical study included 64 patients with POAG requiring first cataract or glaucoma surgery between September 20, 2021 (Ethics Committee approval date) and August 31, 2023 and 64 control patients scheduled for cataract surgery, who met the eligibility criteria described below.

The following common inclusion criteria were used for both participants with cataract and POAG: (1) age of 50 years or older at enrollment; (2) initial surgery; and (3) an eye schedule to be operated on first if both eyes were eligible. The following common exclusion criteria were used for both cataract and POAG subjects: (1) presence of intraocular or optic nerve diseases other than cataract (or POAG in the case of POAG); (2) any history of intraocular surgery, including laser treatment in the target eye; (3) under medical treatment for hyperlipidemia, diabetes mellitus, mitochondrial encephalomyopathy, sepsis, and autoimmune disease, or chemotherapy for cancer; (4) history of any surgery other than fellow eye surgery within 1 year of enrollment; (5) history of stroke or heart attack; and (6) current smoker or a history of smoking within 1 year of enrollment.

Every POAG patient exhibited gonioscopically open angle, glaucomatous optic disc alterations, and an aberrant visual field, as explained below. Glaucomatous optic disc changes were characterized using criteria that included neuroretinal rim damage, an elevated cup-to-disc ratio, rim thinning, and notches with or without retinal nerve fiber bundle defects. Perimetric testing was performed using the Humphrey Visual Field Analyzer (Carl Zeiss Meditec) and 30-2 Swedish Interactive Threshold Algorithm Standard strategy. Tests were considered reliable when the false-positive and false-negative error rates were < 25% and the fixation loss was < 20%. A visual field defect was deemed to exist when at least one of the following conditions was met: two or more contiguous points with a pattern deviation sensitivity loss of P < 0.01, three or more contiguous points with sensitivity loss of P < 0.05 not crossing the horizontal meridian line, a 10-dB difference across the nasal horizontal midline at two or more adjacent locations, and the glaucoma hemifield test outside of the normal limits.

In patients with cataract, slit-lamp microscopy, fundus examination under mydriasis, and optical coherence tomography (OCT) confirmed the absence of organic abnormalities other than cataracts.

Visual field and OCT data were obtained from the results performed within 3 months of patient enrollment.

### Sample size setting

The primary objective of this clinical study was to determine if there was a difference in serum or aqueous humor lactate levels between the cataract and POAG groups. The sample size for this purpose was set as follows.

Previous researches conducted abroad indicated that resting serum lactate levels in 12 NTG patients and 11 normal control subjects were 0.7 ± 0.04 mM and 1.1 ± 0.16 mM (P = 0.04) (Δ = 0.4), respectively^10^, while in 100 POAG patients and 114 cataract patients, these levels were 2.55 ± 0.4 mM and 1.74 ± 0.25 mM (P = 0.03) (Δ = 0.81), respectively^11^. In contrast, aqueous humor lactate levels were 5.81 ± 0.61 mM in POAG patients and 4.71 ± 0.38 mM (P = 0.04) (Δ = 1.1) in cataract patients^11^.

T-test was done to examine the null hypothesis, stating "there is no difference in serum or anterior chamber lactate levels between patients with and without glaucoma." We assumed a minimum difference of 0.4 and a standard deviation of 0.7 (rounded up from the maximum value of 0.61 reported in the previous study^11^). With a two-sided significance level of 2.5% and a detection power of 80%, 60 cases per group were determined to be required. As both serum lactate and aqueous humor lactate were the main endpoints, the significance threshold for the test was set at 0.05/2. After accounting for dropouts, we decided to recruit 70 patients in each group. Enrollment ceased, however, when 64 eligible patients in each group provided suitable intraoperative aqueous humor and blood samples.

### Parameters analyzed

Within 2 months before the day before surgery, the following data were collected: medical history, height, weight, and BMI [defined as weight (kg) divided by height (m^2^)], systolic and diastolic blood pressure in the upper arm, transcutaneous oxygen saturation (SpO_2_) in the index finger, casual blood glucose, serum lactate, pyruvate, best corrected visual acuity measured with the Landolt’s ring chart, and refractive error to determine best corrected visual acuity (spherical equivalent), central corneal thickness measured using a specular microscope (Konan Medical, Osaka, Japan), and IOP by Goldmann applanation tonometer. The medical history interview included past medical history, drug and smoking habits, the frequency of at least 30 min of physically demanding activity per week resulting in perspiration, and the time elapsed between the patient’s last meal and blood sample collection. To measure the levels of lactic and pyruvic acid, 0.3 ml of aqueous humor was collected at the beginning of cataract or glaucoma surgery after local anesthesia (2% lidocaine eyedrops or sub-Tenon injection). Special precautions were taken to prevent blood contamination during this process.

### Serum and aqueous humor lactate and pyruvate measurements

Serum and aqueous humor lactate and pyruvate concentrations were determined using a kit manufactured by Minaris Medical Co (Tokyo, Japan), and 1 mL of blood or 0.3 mL of aqueous humor was immediately mixed with an equal volume of 0.8 N perchloric acid upon collection and then centrifuged to remove proteins. For lactate measurement, the supernatant was exposed to lactate oxidase in the presence of oxygen, resulting in pyruvate and hydrogen peroxide production. The hydrogen peroxide was subsequently reacted with 4-aminoantipyrine and the hydrogen donor *N*-ethyl-*N*-(3-methylphenyl)-*N*′-acetylethylenediamine, in the presence of peroxidase. The resulting red–purple quinone dye was colorimetrically measured at 555 nm absorbance using a Hitachi 7180 automated analyzer manufactured by Hitachi High-Tech Corporation (Tokyo, Japan). For pyruvate measurement, the supernatant was reacted with pyruvate oxidase in the presence of phosphoric acid and oxygen, leading to the production of reactive oxygen species. Then, these were reacted with Bis[3-[bis(4-chlorophenyl)methyl]-4-(dimethylamino)phenyl]amine and peroxidase, resulting in the formation of a green, high-sensitivity dye. This dye was colorimetrically measured by absorbance at 755 nm.

### Statistical analysis

Continuous variables underwent normality test using Shapiro–Wilk test. Variables, except for SBP, DBP, mean blood pressure (MBP = (SBP − DBP)/3 + DBP), and BMI, did not exhibit a normal distribution. Data are shown as median and interquartile ranges. To compare each background factor and primary endpoint of serum and aqueous humor lactate and pyruvate concentrations in the POAG and cataract groups, Mann–Whitney U-test was utilized. Best corrected visual acuity was transformed into the logarithm of minimal resolution angle (log MAR). Between-group comparisons of categorical variables were performed with chi-square test.

Single and multiple regression analyses using a forced entry approach were conducted for secondary objectives to explore parameters associated with serum and aqueous humor lactate. Explanatory variables included age, BMI, SpO_2_, exercise, MBP, SE, interval since the last meal to blood collection, and disease (cataract or POAG) when serum lactate was the objective variable. Casual blood glucose at any time was excluded from the explanatory variables due to its involvement in the same metabolic pathway. With aqueous humor lactate as the objective variable, the explanatory variables were age, BMI, casual blood glucose, SpO_2_, exercise, OPP, SE, and disease. Bivariate correlation analysis was conducted using Spearman rank correlation analysis.

The analysis was performed using SPSS (version 25, IBM Japan, Tokyo, Japan), and significance was considered at P < 0.05.

### Generation of a mouse model of elevated IOP induced by microbead injection into the anterior chamber

Wild-type male C57BL/6J mice, aged 20–25 weeks, were obtained from the Jackson Laboratory Japan (Yokohama, Japan) and housed in the Kobe University Animal Breeding Facility. They were kept under a 12-h light/12-h dark cycle at a room temperature of 24 ± 2 °C and had free access to food and water. The mouse model was created following the protocol outlined in our recent study^9^. Specifically, mice were anesthetized with intraperitoneal administration of a ketamine (100 mg/kg) and xylazine (10 mg/kg) mixture and the pupil of the left eye was pharmacologically dilated with 0.5% tropicamide and 0.5% phenylephrine hydrochloride mixed ophthalmic solution (Santen Pharmaceutical Co., Osaka, Japan). Then, we injected 6 μL of magnetic microbeads (8 μm diameter, 6 × 10^6^ beads/mL in PBS; COMPEL™ Magnetic, COOH modified, UMGB003, Bangs Laboratories, Inc, Fishers, IN, USA) along with 4 μL of air bubbles. To direct the beads into the anterior chamber angle and obstruct the trabecular meshwork, we used a neodymium magnet (TN10-6T1RA-1P, TRUSCO NAKAYAMA CORPORATION, Tokyo, Japan). The untreated right eye served as a control. Additionally, a separate group of control mice received an injection of 6 μL of PBS and 4 μL of air bubble into the anterior chamber of the left eye. After the procedure, the mice were allowed to recover on a heating pad, and Tarivid Ophthalmic ointment (Santen Pharmaceutical Co.) was applied to their eyes.

IOP measurements were taken on the day following treatment, as well as on days 7, 14, and 28. IOP was measured in awake mice using a rebound tonometer (TonoLab; Tiolat, Helsinki, Finland)^9^. During each session, IOP was recorded six times, and internal software was used to eliminate the highest and lowest measurements, averaging the remaining values. This was repeated over five consecutive sessions, and the average IOP values were averaged to determine the IOP at specific time points.

Mice were sacrificed 2 (n = 3) and 4 (n = 3) weeks after microbead injection and 4 weeks after air injection (n = 4) as controls. The retinas were dissected, immediately frozen, and stored for metabolomic analysis as described below. The previous study confirmed that IOP elevation by this approach significantly reduced RGC density at 2- and 4-week time points.

### Metabolomics profiling using gas chromatography/mass spectrometry

Metabolomics profiling was conducted following the previously outlined instructions^13^. The extraction process for low-molecular-weight metabolites involved mixing frozen tissue with 250 μL of a methanol–water–chloroform solvent mixture (2.5:1:1, v/v/v) that included 10 μL of an aqueous solution of sinapinic acid (0.5 mg/mL in distilled water; Sigma Aldrich Tokyo, Japan) as an internal standard. After shaking at 1200 rpm and 37 °C for 30 min, the mixture was centrifuged for 3 min at 4 °C at 22,000*g*. Subsequently, 200 μL of distilled water was added to 225 μL of supernatant, and the mixture underwent another centrifugation for 3 min at 4 °C at 22,000*g*. Following this step, 250 μL of the supernatant was transferred to a fresh tube and lyophilized using a freeze-dryer. The lyophilized samples were combined with 20 μL of methoxyamine hydrochloride (20 mg/mL in pyridine; Sigma-Aldrich) and shaken at 1200 rpm for 90 min at 30 °C to perform oximation. Afterward, the mixture was centrifuged at 22,000*g* for 5 min at 4 °C after adding 10 μL of *N*-methyl-*N*-trimethylsilyl-trifluoroacetamide (GL Science, Tokyo, Japan) for derivatization. The mixture was then incubated at 1200 rpm for 30 min at 37 °C, and gas chromatography/mass spectrometry (GC/MS) analysis was performed on this supernatant.

Utilizing GCMS-QP2010 Ultra (Shimadzu Co, Kyoto, Japan) with fused silica capillary column (CP-SIL 8 CB low bleed/MS; 30 m × 0.25 mm inner diameter, 0.25 μm film thickness; Agilent Co, Palo Alto, CA, USA), GC/MS analysis was performed in the Integrated Center for Mass Spectrometry, Kobe University Graduate School of Medicine. The column’s helium gas flow rate was 39.0 cm/s, and the front inlet temperature was 230 °C. The column was maintained at 80 °C for 2 min isothermally; then, the temperature was increased to 330 °C at 15 °C/min and maintained for 6 min isothermally. The transfer line and ion-source temperatures were 250 °C and 200 °C, respectively. Twenty scans were acquired over the 85–500 m/z mass range using the Advanced Scanning Speed Protocol (ASSP, Shimadzu Co).

MS-DIAL software^14^ was used to perform peak detection, alignment, and identification after exporting MS data in netCDF format. A library of 438 metabolites is available for annotation in MS-DIAL. The quantification process involved the computation of each ion’s peak height, normalization using the peak height of sinapinic acid as the internal standard, and tissue weight adjustment.

### Metabolite analyses

Metabolite data exported from the MS-DIAL software in CSV format were uploaded to MetaboAnalyst 5.0 (https://www.metaboanalyst.ca/home.xhtml), where sample data were normalized by log transformation and autoscaling. Since GC/MS was completed on the same day in the present study, the inspection date block was not adjusted. To achieve natural interactions between samples (grouping, clustering, and outlier detection) and quality control, principal component analysis was employed^15,16^. To determine the 25 metabolites with the biggest difference among the groups, individual and group stratified clustering analyses were done. Metabolite analysis was done to distinguish the 4-week air-injected (control), 2-week microbead-injected, and 4-week microbead-injected groups using PLSDA and VIP selection methods^15,17^. Metabolite alterations with VIP scores higher than one were of particular interest^18^. ANOVA and the Tukey–Kramer test were used to compare the three groups, with P < 0.05 being regarded as significant.

### Ethical approval

The multi-institutional cross-sectional study aimed to investigate differences in serum and aqueous humor lactate and pyruvate concentrations between POAG and cataract patients and their association with patient background factors. The participating institutions included Grace Eye Clinic, Kanamori Eye Clinic, Maeda Eye Clinic, Hiroshima University, Shimane University, and Kobe University in Japan. The study was carried out in strict adherence to the 2013 revised Declaration of Helsinki and the Ethical Guidelines for Life Science and Medical Research Involving Human Subjects issued by the Japanese Ministry of Education, Culture, Sports, Science and Technology, Ministry of Health, Labor and Welfare, and Ministry of Economy, Trade and Industry. It received approval from the Medical Ethics Committee of the Graduate School of Medicine, Kobe University (B210156). All participants provided written informed consent after a comprehensive explanation of the study.

Retinal metabolome analysis using a mouse model with high IOP was performed at Kobe University. Animal experiments were approved by the Animal Care Committee of the Kobe University Graduate School of Medicine (No. P200708) and conducted in accordance with the guidelines stipulated in the Association for Research in Vision and Ophthalmology Resolution on Care and Use of Laboratory Animals Guidelines and the ARRIVE guidelines.

## Data Availability

The datasets generated and/or analyzed during the current study are available from M. Nakamura, the corresponding author, on reasonable request.
